# Nanobiosensors Based on Chemically Modified AFM Probes: A Useful Tool for Metsulfuron-Methyl Detection

**DOI:** 10.3390/s130201477

**Published:** 2013-01-24

**Authors:** Aline C.N. da Silva, Daiana K. Deda, Alessandra L. da Róz, Rogilene A. Prado, Camila C. Carvalho, Vadim Viviani, Fabio L. Leite

**Affiliations:** 1 Multidisciplinary Laboratory of Nanoneurobiophysics, Nanoneurobiophysics Research Group, Department of Physics, Chemistry and Mathematics, Federal University of São Carlos, CP 3031, Sorocaba-SP, 18052-780, Brazil; E-Mails: aline_cristines@hotmail.com (A.C.N.S.); alroz_pessoal@yahoo.com.br (A.L.R.); 2 Laboratory of Biochemistry and Biotechnology of Bioluminescence, Department of Physics, Chemistry and Mathematics, Federal University of São Carlos, CP 3031, Sorocaba-SP, 18052-780, Brazil; E-Mails: pradorogilene@yahoo.com.br (R.A.P.); camilacarvalho.bio08@gmail.com (C.C.C.); viviani@ufscar.br (V.V.)

**Keywords:** enzymes, herbicides, nanobiosensors, atomic force microscopy, atomic force spectroscopy, acetolactate synthase, chemical force microscopy

## Abstract

The use of agrochemicals has increased considerably in recent years, and consequently, there has been increased exposure of ecosystems and human populations to these highly toxic compounds. The study and development of methodologies to detect these substances with greater sensitivity has become extremely relevant. This article describes, for the first time, the use of atomic force spectroscopy (AFS) in the detection of enzyme-inhibiting herbicides. A nanobiosensor based on an atomic force microscopy (AFM) tip functionalised with the acetolactate synthase (ALS) enzyme was developed and characterised. The herbicide metsulfuron-methyl, an ALS inhibitor, was successfully detected through the acquisition of force curves using this biosensor. The adhesion force values were considerably higher when the biosensor was used. An increase of ∼250% was achieved relative to the adhesion force using an unfunctionalised AFM tip. This considerable increase was the result of a specific interaction between the enzyme and the herbicide, which was primarily responsible for the efficiency of the nanobiosensor. These results indicate that this methodology is promising for the detection of herbicides, pesticides, and other environmental contaminants.

## 1. Introduction

The use of pesticides and fertilisers has increased considerably in recent years due to the elevated global demand for food. As a result, there has also been an increase in the exposure of ecosystems to these highly toxic compounds [1–4]. This increased exposure justifies the many studies being carried out to assess the cytotoxic and genotoxic effects of these substances [5–9]. It has been reported that exposure to herbicides, such as paraquat and atrazine, may cause extensive DNA damage [10–12]. Due to their physicochemical characteristics, such as the lipophilic nature and high rate of absorption by organic matter, combined with the high retention time in nature, pesticides are considered hazardous to organisms of all trophic levels. Studies have shown that the exposure of animals to herbicides causes changes in their development and in their immune and reproductive systems. Human exposure to these compounds can cause headaches, endocrine disorders, and even cancer [13–18].

The traditional methods employed for agrochemical detection usually involve chromatographic techniques with ultraviolet (UV) [10,11] or mass spectrometry detection [12]. However, many pesticides currently used are not amenable to detection by chromatographic techniques, at least not at concentrations low enough to be within the safe limits for flora, fauna, and human health [13]. Thus, new methods for sample preparation and analysis, especially for pesticide residues in environmental samples, have been developed in recent years, including the use of sensors and biosensors [19–28].

Studies have highlighted the use of chemically modified tips and micro-cantilevers for the development of nanosensors and nanobiosensors, which expand upon the applications of atomic force microscopy (AFM), making AFM a promising technique for detecting different analytes, microorganisms, and proteins [29–35]. The use of functionalised cantilevers in AFM imaging (chemical force microscopy—CFM) [29,30,36] has made it possible to detect specific intermolecular interactions, and thus, functional groups on a substrate at the micro- and nanometre scale can be mapped. Additionally, CFM can quantify the strength of a variety of interactions, including non-covalent chemical and biological interactions, as a function of the tip-sample distance [37,38]. The use of nanosensors in AFM to quantify the forces between the tip and the sample, which can provide additional information in the form of topographic images, is known as atomic force spectroscopy (AFS). The combination of sensors/biosensors with AFS allows the development of highly sensitive and selective devices [39–44].

When AFS is used for agrochemical analysis, an alternative method for conferring selectivity to a sensor is based on the mechanism of action of the pesticide on the target plant because each pesticide has a unique mechanism of action that usually involves specific binding to a biomolecule. This relevant alternative has been explored by our research group. We have used enzymes to functionalise the AFM cantilevers and tips, allowing the detection of pesticides, especially enzyme inhibitors.

We have been working with acetolactate synthase (ALS), also known as acetohydroxy acid synthase, which is the first common enzyme in the biosynthetic pathway of branched-chain amino acids. ALS is found in bacteria, yeast, and higher plants [45]. It is the primary target site of action for at least three structurally distinct classes of herbicides: sulfonylureas, imidazolinones, and triazolopyrimidines [46].

Using molecular modelling techniques, we have recently designed a nanobiosensor based on microcantilevers functionalised with acetyl co-enzyme A carboxylase (ACCase) to detect the herbicides diclofop and atrazine. The selectivity of the nanosensor for these herbicides was confirmed by semi-empirical calculations and was validated by experimental results [47]. In this work, we studied the detection of the herbicide metsulfuron-methyl, one of the most widely used herbicides in Brazil for post-emergence weed control in wheat fields [48], by employing a new methodology based on the use of AFM tips modified with the enzyme ALS.

## 2. Experimental Section

### 2.1. Expression of Recombinant ALS

The cDNA of ALS (from *Oryza sativa*) inserted into the *Eco*RI sites of the pGEX 2T vector was kindly provided by Dr. Tsutomu Shimizu from the Life Science Research Institute, Shizuoka, Japan. The plasmid was used to transform *E. coli* BL21-DE3 cells. The colonies obtained after transformation were grown in 500–1,000 mL of LB medium containing 100 μg/mL ampicillin at 37 °C to OD 600 = 0.4 and then induced with IPTG at 22 °C for 3 to 4 h. The cells were harvested by centrifugation at 2,500 g (≈4,400 rpm) for 15 min and resuspended in 1X PBS buffer containing complete protease inhibitors (Roche), freeze-thawed three times in dry-ice, and centrifuged at 15,000 g for 15 min at 4 °C. The ALS-containing supernatant, referred to as the crude extract, was used for the acetolactate synthase assay, protein determination, and cantilever functionalisation.

#### 2.1.1. Acetolactate Synthase Activity and Herbicide Inhibition Assays

ALS activity was assayed according to the methods of Kawai and coworkers [49] in a 1 mL reaction mixture containing 20 mM sodium pyruvate, 0.5 mM thiamine pyrophosphate, 0.5 mM MgCl_2_, 10 μM flavin adenine dinucleotide, and 20 mM potassium phosphate buffer (pH 7.5). After the enzyme solution was added, the reaction mixture was incubated at 37 °C for 30 min. Next, the reaction was stopped by the addition of 100 μL of 6 M H_2_SO_4_ and heated at 60 °C for 15 min to convert the acetolactate to acetoin. Then, 1 mL of 0.5% (w/v) creatin and 1 mL of 5% α-naphthol (w/v) dissolved in 2.5 M sodium hydroxide were added to the mixture. The acetoin formation was then determined by spectrophotometric analysis at 525 nm and by colour comparison. A reaction without substrate (sodium pyruvate) was used as the blank. As a control for endogenous expression of ALS in non-recombinant *E. coli*, crude extracts of bacteria lacking the ALS cDNA containing plasmid were used.

The inhibition of the recombinant ALS by metsulfuron-methyl was examined. The stock solution of metsulfuron-methyl (2 mM) was dissolved in methanol, and working solutions were further dissolved in pure water. Different concentrations of metsulfuron-methyl were added to the reaction mixture before the addition of ALS containing crude extracts.

### 2.2. Chemical Functionalisation of Tips and Substrates

The functionalisation of the tips (silicon nitride) and substrates (muscovite mica) was carried out by adapting the method described by Wang and collaborators [50]. After the tips and substrates had been cleaned in a UV chamber (240 nm; ProCleaner, UV.PC.220, Bioforce) [51], the functionalisation process was initiated by the gaseous evaporation of 3-aminopropyltriethoxysilane (APTES) in the presence of triethylamine. Then, a small aliquot of a glutaraldehyde solution (1 × 10^−3^ M) was added, followed by the addition of the ALS enzyme-enriched extract (0.200 mg/mL, ∼1.3 × 10^−6^ M) to one tip and 1 mM ALS-inhibiting herbicide metsulfuron-methyl (in methanol) to the substrate. The tips and substrates were washed three times with small aliquots of deionized water to remove the excess of unbound enzyme and herbicide, respectively. The tips were also evaluated by scanning electron microscopy (data not shown), where the images confirmed their integrity after the functionalisation process. All reagents used, except the ALS enzyme, were purchased from Sigma.

### 2.3. Fourier Transform Infrared Spectroscopy

FTIR spectra were recorded using a Nicolet-IR200 (FTIR-410) Thermo Scientific FTIR spectrometer (Jasco) using the attenuated total reflectance (ATR) technique. Because of the small dimensions of the biosensor, the functionalised tip was reproduced on a macroscopic scale using a plate of silicon nitride functionalised according to the same procedure described in Section 2.2.

### 2.4. Atomic Force Spectroscopy (AFS)

The force spectroscopy experiments were performed with an AFM Multimode-VS System with the PicoForce package (dedicated to force spectroscopy). The AFM tip employed in the determination of the force curves was made of silicon nitride (V-shaped, model NP-10 by Veeco) with a nominal spring constant (K) of 0.12 N/m. However, due to the considerable variations that can occur between the nominal and real values of the spring constant, each AFM tip was subjected to a calibration procedure using the thermal noise method [52]. The force curves were obtained at 25 °C and a relative humidity between 30% and 40% using muscovite mica as the substrate. To analyze dilute solutions, the *drop casting* method was used. This method forms a bubble of solution on top of the mica surface. Muscovite mica was chosen because it is molecularly smooth and because it can be cleaved immediately prior to use, thereby minimising the need for further cleaning. Two methods for herbicide detection were used: (i) detection using tips functionalised with the ALS enzyme, and (ii) detection using unfunctionalised tips. Force curves were obtained by measuring the adhesion force values for various substrates, at different points on each substrate, and using different tips to evaluate the efficiency of the nanobiosensor.

## 3. Results and Discussion

### 3.1. Evaluation of the Enzymatic Activity and Inhibition of ALS by Metsulfuron-Methyl

**Figure 1.**
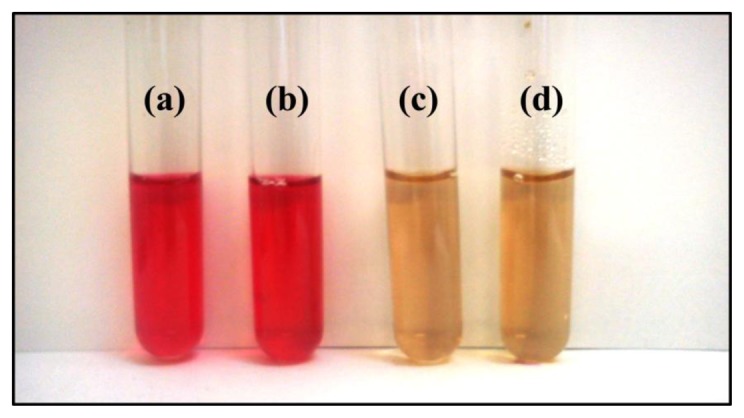
Colorimetric enzyme assay for recombinant ALS. The distinct colour reveals the presence (a,b) or absence of acetoin (c,d). (**a**) Reaction mixture containing freshly extracted ALS; (**b**) ALS 24 h after extraction; (**c**) control mixture without substrate; and (**d**) control mixture containing the crude extract of *E. coli* lacking the ALS cDNA.

ALS is not found in humans, and it is thus an effective target for herbicides. Therefore, this enzyme is attractive for addressing numerous goals of modern herbicide research, including the development of techniques for detecting herbicides. The catalytic activity of acetolactate synthase was spectrophotometrically assessed based on the formation of acetoin upon the decarboxylation of the enzymatically formed acetolactate with creatine and naphthol. Figure 1 shows reaction mixtures that produced acetoin (red) or did not produce acetoin (yellow). The ALS in these preparations was stable, retaining catalytic activity for over 24 h [49].

**Figure 2.**
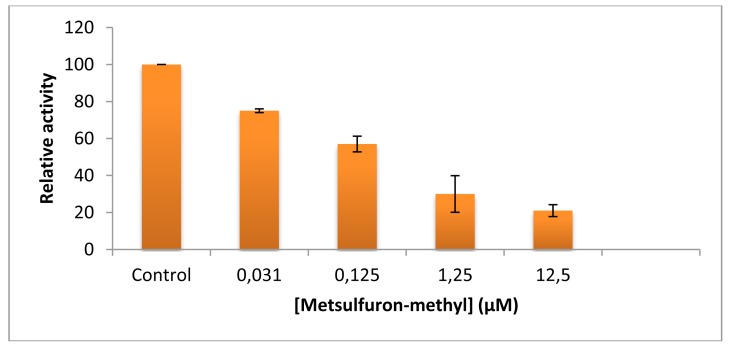
Effect of metsulfuron-methyl on the activity of recombinant ALS.

As shown from the literature [53], metsulfuron-methyl is a very strong inhibitor of ALS. In our ALS assays, the herbicide concentration required for 50% inhibition was 0.125 uM indicating, as expected, the high sensitivity of ALS to inhibition by this kind of herbicide (Figure 2). Therefore, the herbicide concentration used in AFM assays (1 mM) was suitable for qualitative determination of metsulforon-methyl by ALS.

### 3.2. Chemical Functionalisation of Tips and Substrates

**Figure 3.**
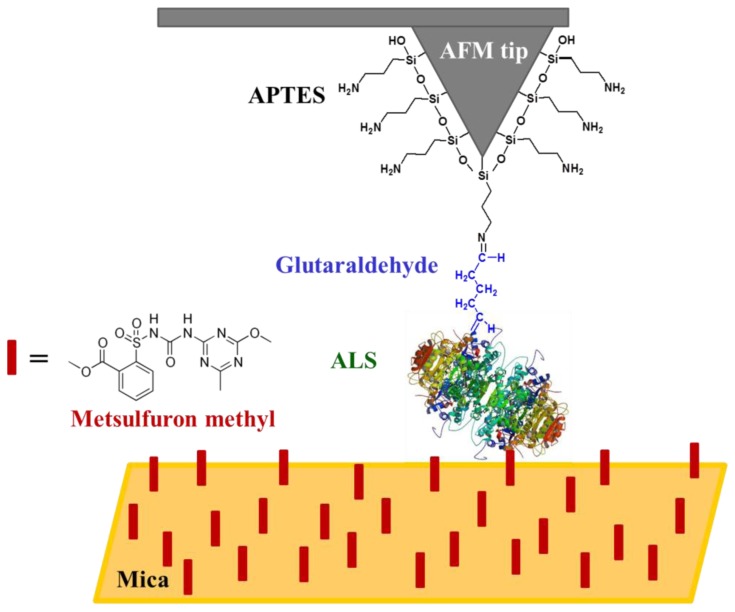
Schematic illustrating the functionalisation of the silicon nitride tip and the detection of metsulfuron-methyl herbicide on the mica surface.

A schematic representation of the functionalised tip used for the metsulfuron-methyl detection is shown in Figure 3. The binding of the APTES to Si_3_N_4_ is made such a way that the other end of the molecule, the amino group, is free to interact with glutaraldehyde via the formation of a Schiff base [50]. The binding of the ALS enzyme (from ALS-enriched extracts) to glutaraldehyde also occurs, most likely via the formation of another Schiff base due to a reaction between the aldehyde and an -NH_2_ group present in the ALS enzyme. In this reaction, the nucleophilic nitrogen of the amino group displaces the oxygen from the aldehyde, which causes the loss of one water molecule and forming a C=N bond [50]. The substrate functionalisation occurred in a similar manner to that described for the tip.

### 3.3. Fourier Transform Infrared Spectroscopy

**Figure 4.**
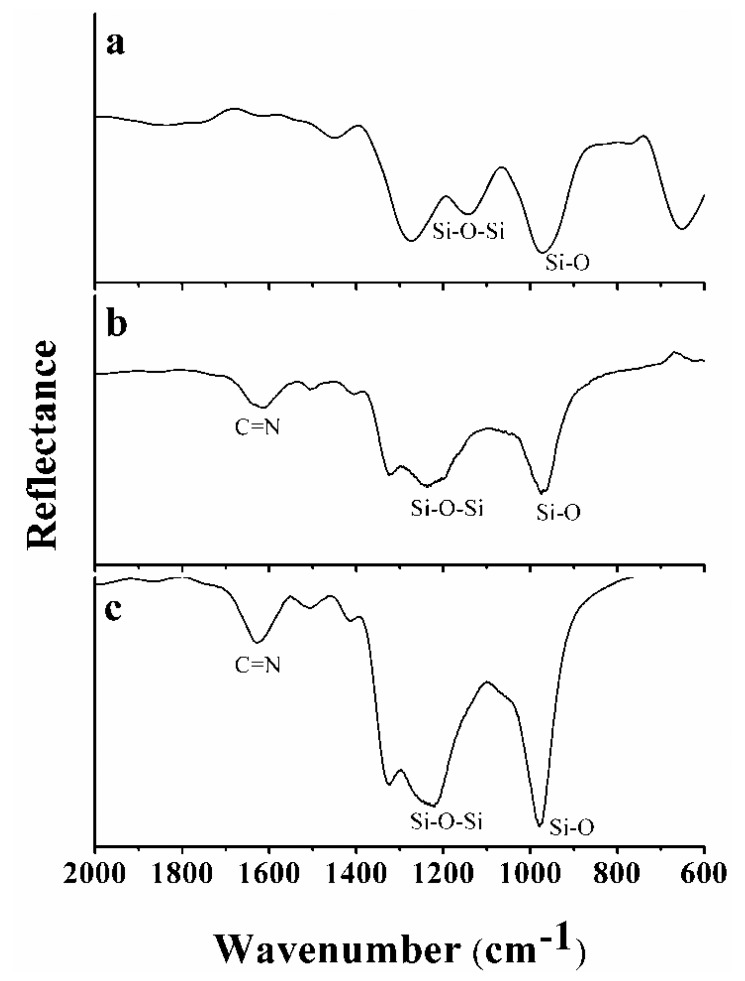
FTIR spectra of (**a**) Si_3_N_4_, (**b**) Si_3_N_4_ reacted with APTES and glutaraldehyde and (**c**) Si_3_N_4_ functionalised with ALS.

The functionalisation components (Si_3_N_4_, APTES, glutaraldehyde and ALS) were characterised by FTIR [54]. Figure 4 shows the FTIR spectra for Si_3_N_4_ (Figure 4(a)), Si_3_N_4_ reacted with APTES and glutaraldehyde (Figure 4(b)), and Si_3_N_4_ after functionalisation with the ALS enzyme-enriched extracts (Figure 4(c)).

The main characteristics of the FTIR spectrum of Si_3_N_4_ (Figure 4(a)) are two peaks: (i) 976 cm^−1^, associated with Si-O stretching, and (ii) 1,200 cm^−1^, associated with the Si-O-Si stretching vibration mode [55]. The presence of a peak at 1,623 cm^−1^, indicating Schiff base formation, in the spectrum (Figure 4(b)) demonstrated that there was an interaction between APTES and glutaraldehyde, [50,56]. The same Schiff base formation occurred between glutaraldehyde and the proteins present in the ALS-enriched extracts, as shown in Figure 4(c), as the result of a reaction between the aldehyde group of glutaraldehyde and an -NH_2_ group in ALS or other proteins. Because the extracts were enriched with ALS, most of the interactions involved ALS and the herbicide, but non-specific interactions between the herbicide and other proteins, e.g., BSA, could also be detected (bacteria extracts). The results showed that the functionalisation process was effective.

### 3.4. Evaluation of the Interaction between the Enzyme and the Herbicide

**Figure 5.**
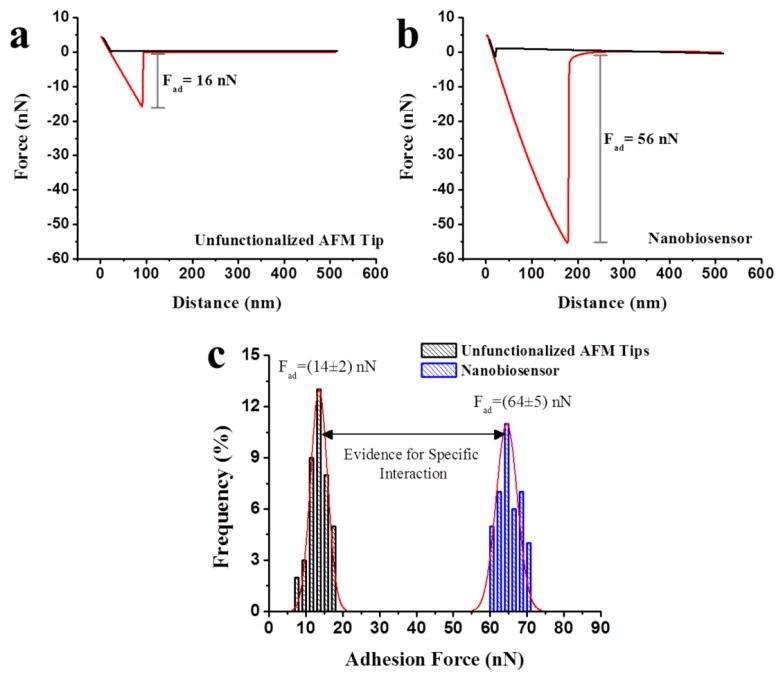
Example of typical force curves obtained using (**a**) an unfunctionalised tip, (**b**) a functionalised tip (nanobiosensor) and (**c**) histograms obtained for the values of the adhesion force (n = 5,000) when using unfunctionalised tips and nanobiosensors to analyse substrates covered with the herbicide metsulfuron-methyl.

Figure 5(a,b) show typical curves obtained when measuring the adhesion force between the herbicide metsulfuron-methyl and an unfunctionalised tip and a functionalised tip (nanobiosensor, with the modified substrate), respectively. The adhesion force was determined using the force curves by analysing the point of maximum deflection of the cantilever before total detachment from the surface (the region indicated by the grey lines in Figure 5(a,b)). The adhesion value when the nanobiosensor was employed was ∼3.5-fold greater than the value obtained with the unfunctionalised tip. This significant difference highlighted the importance of the chemical modification because the functionalisation allowed specific binding between the tip and the substrate, greatly increasing the adhesion force and making the nanobiosensor extremely sensitive. The importance of the specific interactions to the detection efficiency of nanosensors have been reported previously in studies involving cantilevers functionalised with antibodies specific for some herbicides [57–60]. The adhesion force between *bacterial extracts* and metsulfuron-methyl was not significant (≤16 nN); thus, the interaction between the functionalised tip and the herbicide was purely specific.

Figure 5(c) shows the histograms obtained for more than 5,000 consecutive force curves, and these curves show an increase in the adhesion force when employing the nanobiosensor. It was observed adhesion force values of 14 ± 2 nN and 64 ± 5 nN when the unfunctionalised tip and the nanobiosensor were employed, respectively. Low deviations were observed for these two measurements (<15%) due to the high specificity of the nanobiosensor. Additionally, the deviation obtained for the 5,000 measurements was considered to be satisfactory because it was an indication of the integrity of the nanobiosensor, which retained its detection capacity even after being used to generate numerous force curves.

**Figure 6.**
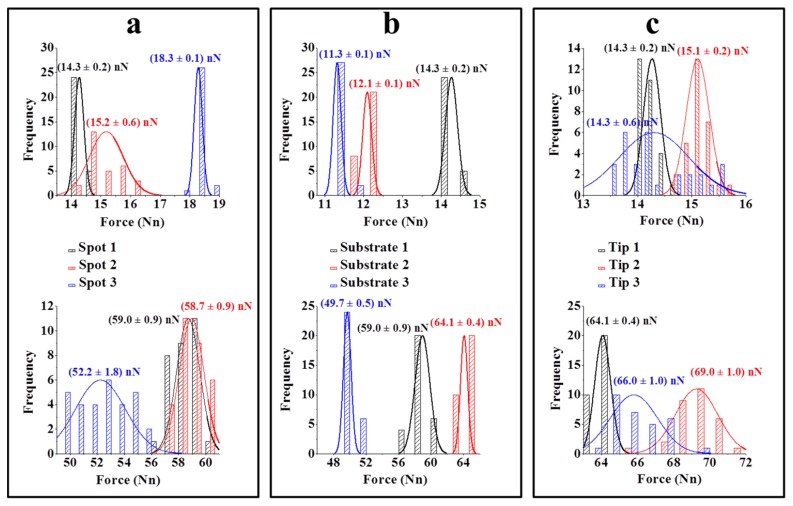
Comparative graphs of the force curve measurements taken using unfunctionalised (above) and functionalised (below) AFM tips (**a**) at three different points on the same substrate, (**b**) on three substrates with herbicides (triplicate), and (**c**) using three different tips (silicon nitride).

The variation in the adhesion force measurements was also evaluated on the sample surface. Force curves were obtained at three different points on each substrate modified with the herbicide (Figure 6(a)). Negligible variation was observed between the mean values for each point of the substrate, with variations of approximately 12% for the unfunctionalised tip and 7% for the tip modified with ALS. Similar results were obtained in the analysis of the force curves obtained for different substrates (Figure 6(b)) and different tips (Figure 6(c)). These results indicate the reproducibility of the method, especially in respect to the process of modifying the substrate and tips which may be considered homogeneous. As a result, small variations in the adhesion force values observed maybe can be promising for the quantitative determination of this herbicide.

## 4. Conclusions

The use of chemically modified cantilevers has proven to be a promising alternative for the detection of enzyme-inhibiting herbicides, allowing the use of AFM also as a qualitative detection approach. By combining tips functionalised with ALS and force curve measurements, it was possible to detect, for the first time, the herbicide metsulfuron-methyl. The comparison of the adhesion force values between the substrate covered with the herbicide and functionalised (nanobiosensor) and unfunctionalised (bare) tips highlighted the potential of this technique and of the use of enzymatic biosensors for the detection of agrochemicals. The adhesion force measured with the nanobiosensor was ∼250% greater than that measured with the unfunctionalised tip. Thus, the results indicate that the development of sensors/biosensors based on specific interactions is an excellent alternative to provide greater sensitivity and selectivity, making AFS more effective in detecting enzyme-inhibiting herbicides.
